# The influence of information status on pronoun resolution in Mandarin Chinese: evidence from ERPs

**DOI:** 10.3389/fpsyg.2015.00873

**Published:** 2015-07-06

**Authors:** Xiaodong Xu

**Affiliations:** School of Foreign Languages and Cultures, Nanjing Normal UniversityNanjing, China

**Keywords:** information status, topic, order of mention, pronoun resolution, ERP

## Abstract

The purpose of this study is to shed light on the neural mechanisms underlying the modulation of pronoun resolution processes by the information status of the antecedent. Information status was manipulated by using a structurally based constraint (e.g., order of mention) as well as a pragmatically based constraint (i.e., topichood). We found that the pronouns referring to topic entities [the initial noun phrase (NP) in Subject–Object–Verb (SOV) structure in Experiment 1 and OSV structure in Experiment 2] elicited attenuated P600 responses compared to the pronouns referring to non-topic entities (the initial NP in SVO structure or the second NP in OSV structure in both experiments) when potential interference from structural constraints was controlled. The linear structural constraint, namely the order of mention, had no clear influence on the P600 effect when the syntactic structural constraint was held constant (i.e., when both entities were syntactic subjects), regardless of whether one (Experiment 1) or two (Experiment 2) animate antecedents were present. These findings suggest that pragmatically encoded features such as topichood and givenness can be processed separately from structural constraints such as order of mention to promote the salient status of a referent and thereby facilitate pronoun interpretation.

## Introduction

During communication, language users want their utterances/sentences to be well formed, not only syntactically but also informationally. The information status of a referent can be realized by structural manifestations such as linear order (e.g., whether it is the first or the second noun in a sentence: surface structure) and syntactic role (e.g., whether it is the subject or object of a sentence: deep structure), it can also be realized by pragmatic/discursive considerations such as the given-new distinction, topic-focus, or topic-comment distinction (Arnold et al., 2013). In particular, the pragmatic aspect of information status can be assigned through structural manipulations such as word order variation, especially for languages with flexible word orders (e.g., Chinese, Finnish). For example, in a Chinese sentence using the non-canonical Subject–Object–Verb (SOV) word order, the initial noun phrase (NP) is typically assigned topic-related features such as aboutness and givenness, which are not typically assigned in the more canonical SVO structure. The information status of an entity influences its salience in discourse and to some extent determines whether and how it will be referred in subsequent discourse. The purpose of the present study is to shed light on the neural mechanisms underlying pronoun resolution, and particularly the aspects of pronoun resolution that take the antecedent’s information status into account.

A considerable number of studies have found that entities realized in subject position are more likely to be interpreted as the referent of a subsequent pronoun than entities realized in non-subject positions (e.g., object position), the so-called subject preference of pronoun resolution (Crawley et al., 1990; Gordon et al., 1993; Gelormini-Lezama and Almor, 2011; Kaiser, 2011). Other studies have shown that the first-mentioned entity in the preceding clause/sentence is more likely to be interpreted as the referent of a later pronoun than the second-mentioned entity (Gernsbacher and Hargreaves, 1988; Arnold et al., 2000). However, although these studies have provided evidence for the importance of structural constraints on pronoun resolution, they may not have disentangled the role of subject preference (a syntactic constraint/surface structural constraint) and first-mention preference (a linear constraint/deep structural constraint), since the subject is also structurally the first referent in languages tested in most of these studies. In order to distinguish between first-mention preference and subject preference, Järvikivi et al. (2005) turned to Finnish, a language with free word order, which not only has SVO structure but also allows OVS structure when the object is topicalized to convey given information. Evidence from reading times showed distinct effects of syntactic role (i.e., subject preference) and order of mention (first mention preference), but no interaction between them, suggesting that although both order of mention and syntactic role play important roles in determining the interpretation of a pronoun, they work in an independent manner—that is, one can not override the other.

Pronoun resolution is also modulated by discursive/pragmatic information status, such as topic and focus. The *topic* of an utterance is regarded as what the utterance is about, denoting shared information (i.e., givenness) between the speaker and addressee, whereas the *focus* presents information that is new and unpredictable for the addressee (i.e., newness; Reinhart, 1982; Li and Thompson, 1989; Colonna et al., 2012). Although the discourse functions of topic and focus devices may be different, both devices appear to render referents more prominent in discourse (Almor, 1999; Cowles et al., 2007b; Foraker and McElree, 2007; Colonna et al., 2012). According to the prominence account of anaphora interpretation (e.g., the Accessibility theory: Ariel, 1988, 1990; and the Givenness hierarchy: Gundel et al., 1993), pronouns have a tendency to be resolved as referring to more prominent antecedents in discourse. This is because such referents are more active in memory and thus more available to pronoun resolution processes when a pronoun is encountered (Foraker and McElree, 2007). According to this account, topic and focus devices should have similar effects on the resolution of a pronoun, since both can make an antecedent’s representation more active in the discourse model.

In addition, it should be noted that, although pragmatically encoded information status such as topichood is different from structurally based information status such as syntactic role and order of mention, these two sources of constraints often overlap, since the pragmatic status of a referent can be realized by varying the structural sequence (e.g., from SVO order to SOV order). Considering Järvikivi et al.𠄙s (2005) study as an example, the grammatical object does not necessarily function as a topic when it appears in SVO structure, but does (and is thus more prominent in the discourse) when it appears in OVS structure. Thus, effects of order of mention and topichood would have been difficult to distinguish.

The processing of pronoun–antecedent relations has also been investigated using event-related brain potentials (ERPs; Cowles et al., 2007a; Hirotani and Schumacher, 2011; Hung and Schumacher, 2012). According to one psycholinguistic model of anaphora resolution, pronoun/anaphora resolution can be differentiated into a bonding stage and a resolution stage (Garrod and Sanford, 1994; Garrod and Terras, 2000). In the bonding stage, candidate antecedents are retrieved and the retrieval (or linking) is constrained by morpho-syntactic rules (e.g., gender, number, case agreement). In the resolution stage, the appropriate antecedent is selected and integrated with the pronoun as well as the whole discourse. These two processing stages are associated with different ERP components. The bonding stage has been revealed to be related with some early manifestations (e.g., early negativities) in languages with morpho-syntactic agreement features (Demestre et al., 1999; Lamers et al., 2006, 2008), although the early modulation was not consistently observed in languages lacking such an agreement system (Qiu et al., 2012; Xu et al., 2013). Nonetheless, compared to the inconsistent findings in bonding processing, resolution processing is generally manifested by a late positivity (P600) regardless of cross-linguistic variations (Osterhout and Mobley, 1995; Lamers et al., 2006, 2008; Qiu et al., 2012; Silva-Pereyra et al., 2012), reflecting the process of integrating the referential relation with the discourse context.

A number of ERP investigations have shown a P600 when there is an agreement mismatch (e.g., gender, number, person) between the pronoun and its antecedent (Osterhout and Mobley, 1995; Molinaro et al., 2008b; Xu et al., 2013), or even in situations where a pronoun is coreferential with a less accessible referent rather than a more accessible one (van Berkum et al., 2007; Qiu et al., 2012). In particular, the P600 can be divided into early and later subprocesses (Barber and Carreiras, 2005; Molinaro et al., 2008a, 2011a; Xu et al., 2013). According to previous ERP studies concerning pronoun/anaphora resolution (e.g., Silva-Pereyra et al., 2012), the early P600 appears to be associated with the process of evaluating/testing the link between the pronoun/anaphora and the antecedent, which is modulated by the salient features of the antecedent entity, whereas the late P600 is interpreted as reflecting the effort spent in integrating the pronoun/anaphora-antecedent relation and the context information into a coherent discourse representation. The more demanding the integration processing is, the more enlarged the amplitude of the P600 would be.

For instance, the late positivity elicited by a pronoun is smaller when the pronoun’s antecedent is a topic than when it is a non-topic (Hung and Schumacher, 2012, 2014; Schumacher and Hung, 2012), or when the referent is focused as opposed to unfocused (Xu and Zhou, submitted). However, although the assignment of topic or focus status can increase the prominence of a referent, the cognitive/neural underpinnings underlying these two information structures may not be the identical, since one important distinction between focus and topic is that focus conveys new information (e.g., foreground) whereas topic conveys given information (e.g., background/shared information). Of particular relevance to the present study is an ERP study conducted by Xu and Zhou (under revision), where the authors investigated how topic status and structural constraints interact to affect pronoun resolution. The authors adopted a topic structure (e.g., 1a and 1b) and a non-topic structure (e.g., 2a and 2b). The first noun (NP1, e.g., 王宇/*Wangyu*) occupies sentence-initial position and acts as the sentence-topic in topic structure, but occupies the non-initial (second) position and acts as the subject of subordinate clause in non-topic structure. A gender-marked pronoun can be interpreted to refer to a topic antecedent (i.e., control condition) or a non-topic antecedent (i.e., object) in the topic structure; it can also be interpreted to refer to a subject antecedent (i.e., control condition) or a non-subject antecedent (i.e., object) in the non-topic structure, resulting in four experimental conditions: *topic-continuation* (1a), *topic-shift* (1b), *subject-continuation* (2a), and *subject-shift* (2b). The pronouns referring to non-topic antecedents elicited stronger P600 responses than the ones referring to topic antecedents, whereas no such asymmetry was found for pronouns referring to non-subject versus subject antecedents (in a sentence structure that does not strongly mark the topic). This suggests that topic has a privileged status relative to non-topic entities (e.g., subject, object) in pronoun resolution.

image image *Wangyu because worry about Liwei, so/ he/ insist on/ 24 hours/ keep phone on.* Because Wangyu worries about Liwei, (so) he keeps a 24-hour phone access.

(1a) 王宇因为担心李薇, 所以/他/坚持/二十四小时/开机.

*Wangyu_male_ yinwei danxin Liwei_female_, suoyi/ ta_male_/ jianchi/ ershisixiaoshi/ kaiji*.

Wangyu because worry about Liwei, so/ he/ insist on/ 24 hours/ keep phone on.

Because Wangyu worries about Liwei, (so) he keeps a 24-hour phone access.

(1b) 王宇因为担心李薇, 所以/她/坚持/二十四小时/开机.

Wangyu_male_ yinwei danxin Liwei_female_, suoyi/ ta_female_/ jianchi/ ershisixiaoshi/ kaiji.

Wangyu because worry about Liwei, so/ she/ insist on/ 24 hours/ keep phone on.

Because Wangyu worries about Liwei, (so) she keeps a 24-hour phone access.

(2a) 因为王宇担心李薇, 所以/他/坚持/二十四小时/开机.

Yinwei Wangyu_male_ danxin Liwei_female_, suoyi/ ta_male_/ jianchi/ ershisixiaoshi/ kaiji.

Because Wangyu worry about Liwei, so/ he/ insist on/ 24 hours/ keep phone on.

Because Wangyu worries about Liwei, (so) he keeps a 24-hour phone access.

(2b) 因为王宇担心李薇, 所以/她/坚持/二十四小时/开机.

Yinwei Wangyu_male_ danxin Liwei_female_, suoyi/ ta_female_/ jianchi/ ershisixiaoshi/ kaiji.

Because Wangyu worry about Liwei, so/ she/ insist on/ 24 hours/ keep phone on.

Because Wangyu worries about Liwei, (so) she keeps a 24-hour phone access.

However, these putative differences in topic vs. subject prominence may instead have been due to structural differences. Specifically, in the sentences with topic structure (which showed an asymmetry between topic-referring pronouns and non-topic referring pronouns), the topic noun was sentence-initial and the non-topic noun was not; in sentences without topic structure (which showed no asymmetry), both the subject and non-subject nouns were sentence-medial. Given that previous relevant studies have revealed a processing advantage, as evidenced by a reduced P600 effect (indicating smaller cognitive efforts devoted), for referential expressions in the sentence-initial position over the non-initial (sentence-medial) position (Schumacher and Hung, 2012; Hung and Schumacher, 2014), it is possible that this effect may be a difference between sentence-initial and non-initial expressions, rather than a difference between topic and non-topic expressions. Thus, one aim of the present study is to test the putative topic privilege in a design that controls for sentence position.

Unlike subject-prominent languages such as English, Chinese is a topic-prominent language (Li and Thompson, 1976, 1989; Huang et al., 2009), in which the priority in constructing a sentence is given to the pragmatic pattern of setting up a topic and making comment on it (Xu and Langendoen, 1985; Her, 1991). The topic can be indicated by word order alone, as illustrated in sentence (3), where *Morning newspaper* (早报) is the shared information between the speakers and addressees and is what the rest of the sentence is about.

(3) 早报爷爷读过了. (OSV structure)

Zaobao yeye duguole.

Morning newspaper Grandpa read

The Morning newspaper, Grandpa has read.

A sentence topic can also be morphologically or prosodically marked. For example, topic can be distinguished from the rest of the utterance with a topic marking particle such as *ne*, *a*, *ya*, a pause or a pause particle in Chinese (i.e., a comma; Li and Thompson, 1989; Xu and Liu, 2007). In particular, the use of a comma to separate topic from comment applies not only for the topic-prominent languages like Chinese but also other non-topic-prominent languages like English^1^. As shown in (4), *Zhangsan* (张三) functions as the topic of the sentence, since it is what the remaining part is about and its topic status is set by being placed at the beginning of the sentence and followed by a pause particle, i.e., a comma.

(4) 张三, 李四已经见过了.

Zhangsan, Lisi yijing jianguole.

Zhangsan, Lisi already see.

(As for) Zhangsan, Lisi has seen (him) already.

Chinese has a flexible word order (Li and Thompson, 1976; Huang et al., 2009), which allows not only the canonical SVO structure but also SOV structure and OSV structure. According to Li and Thompson (1976) and others (e.g., LaPolla, 1995), the order in which major constituents of a sentence occur in Chinese is governed to a large extent by consideration of pragmatic or discourse factors (e.g., information status), in contrast to English, which is governed mainly by grammatical relations such as subject and object. Word order variations in Chinese serve primarily to signal pragmatic distinctions such as definite–indefinite, given–new, and topic–comment (Chu, 1998) rather than grammatical relations (e.g., selection constraints, subject–verb agreement) as English word order does. For example, although sentences (5a-c) have identical constituents and propositional meaning, they achieve different communication goals, due to the pragmatic-level differences across structures. While sentence (5a) simply addresses an event that *Grandpa* (爷爷) has read the newspaper, sentence (5b) and (5c) additionally convey pragmatically based implications: when the inanimate noun (i.e., object) *Morning newspaper* in sentence (5a) was moved to a structurally prominent position, namely the sentence-initial position (5b), it was treated as the shared information between speakers and addressees, and the following sentence is about *Morning newspaper*, forming a topic–comment structure where *Morning newspaper* functions as the topic and *Grandpa* as the subject of the sentence. In contrast, if *Morning newspaper* was moved to the front of the verb and after *Grandpa*, it no longer functions as the sentence topic^2^ but rather as the object of the sentence. *Grandpa* in this situation, instead, functions as the topic of the sentence, as it occupies the syntactically prominent position and is what the following contents are about (i.e., aboutness).

(5a) 爷爷读过了早报. (SVO structure)

Yeye duguole zaobao.

Grandpa read Morning newspaper.

Grandpa has read the Morning newspaper.

(5b) 早报爷爷读过了. (OSV structure)

Zaobao yeye duguole.

Morning newspaper Grandpa read.

The Morning newspaper, Grandpa has read.

(5c) 爷爷早报读过了. (SOV structure)

Yeye zaobao duguole.

Grandpa Morning newspaper read.

Grandpa, the Morning newspaper has read.

**FIGURE 1:**
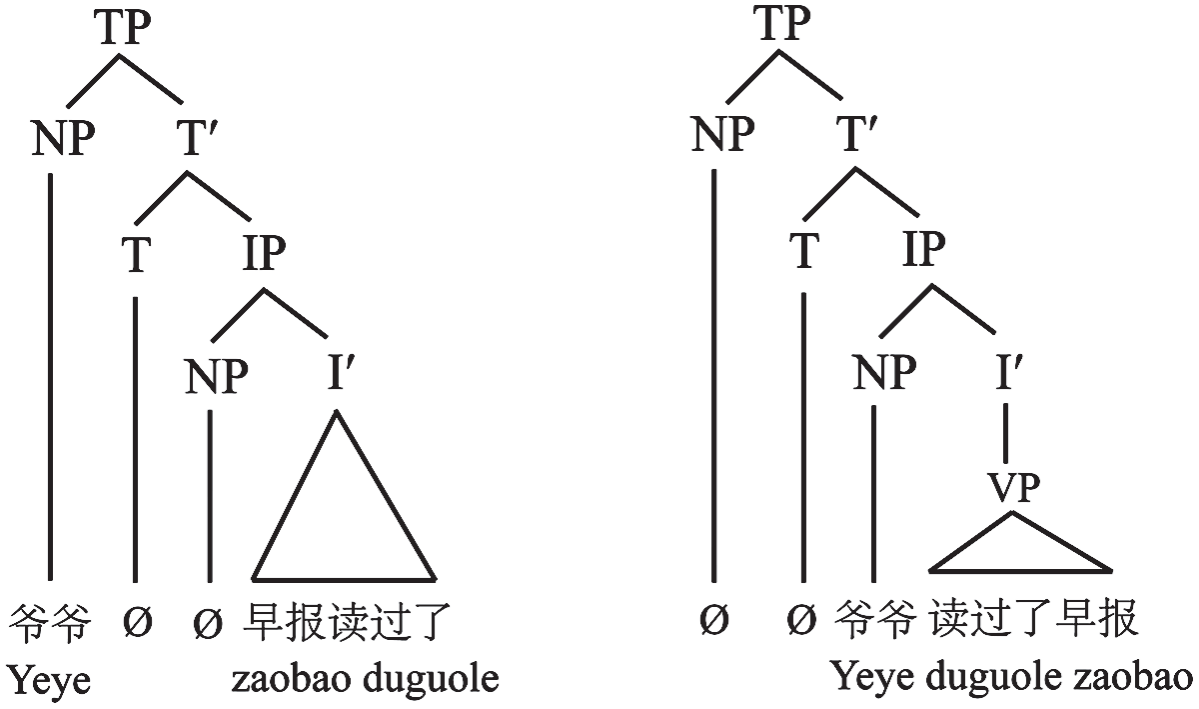
**Syntactic trees for topic structure (left) and SVO structure (right) in Chinese.** VP, verb phrase; NP, noun phrase; I, inflection; IP, inflectional phrase; TP, topic phrase.

Moreover, although *Grandpa* occupies the sentence-initial position in both SVO and SOV structures, distinguishing them from the OSV structure where *Grandpa* occupies the sentence-medial position, *Grandpa* still has different information status in these two subject-initial structures: it (Grandpa) additionally conveys pragmatically-encoded topichood (e.g., aboutness, givenness) in the SOV (i.e., 5c) but not the canonical SVO cases (i.e., 5a). Some generative studies on Chinese topic structures proposed that the initial NP in SOV structure could be interpreted as a sentence topic (i.e., structure topic), in contrast to SVO structure where the initial NP functions as a grammatical subject (Xu and Liu, 2007). As illustrated in **Figure 1**, *Grandpa* (爷爷) functions as a sentence topic in the SOV structure (the left of **Figure 1**), not just because it tells what the rest sentence is about but also because it occurs within the TP^3^ (Topic Phrase; where topic NP occupies [Spec, TP]) but outside the IP^4^ (Inflection Phrase). By contrast, *Grandpa* acts as grammatical subject in the SVO structure (the right of **Figure 1**) because it is located within the IP but outside the VP (Verb Phrase), in other words, it is mainly concerning a “doing” relationship with the following verb in the sentence.

Taken together, as a topic-prominent language with flexible word order, Chinese provides us with a good opportunity to examine the influence of topic status (the pragmatically based information status) and order of mention (the structurally based information status) on pronoun resolution. In this study, we conducted two ERP experiments to shed light on how topichood and order of mention modulate neural signatures of pronoun bonding and resolution. In Experiment 1, we used a topic structure occurring typically in topic-prominent languages such as Chinese; in Experiment 2, a more extensively used topic structure was adopted. Moreover, in order to examine whether the role of information status in pronoun resolution was modulated by the degree of referential competition/complexity, one animate antecedent was presented in Experiment 1 whereas two were presented in Experiment 2.

## Experiment 1

In this experiment, we examined the neural mechanisms underlying pronoun resolution depending on the information status of the antecedent. Specifically, we compared the neural responses evoked by pronouns which refer to animate antecedents within three different constructions: (1) SVO structure (5a), (2) SOV structure (5c), and (3) OSV structure (5b). For the SVO and the SOV structures, the first NP (proper name, e.g., *Grandpa*/爷爷) takes the same thematic role (i.e., agent) and occupies the sentence-initial position. The main difference concerning these two structures is the information status carried by the initial NP, given that the initial NP encodes pragmatically based implications such as givenness and aboutness in the SOV but not the SVO structures. This allowed us to inspect the influence of information status on pronoun resolution without conflating it with thematic/grammatical role and order of mention. Furthermore, the difference in information status carried by the animate NP (i.e., ‘S’) within the SVO vs. the OSV structures comes mainly from the order of mention. By comparing the ERP responses evoked by pronouns referring to antecedents embedded in these three types of constructions, we can contrast the influence of different aspects of information status on pronoun interpretation. Moreover, in order to compare the neural responses generated by information status with those by agreement violation as revealed by previous studies (Osterhout and Mobley, 1995; Osterhout et al., 1997; Xu et al., 2013; Nieuwland, 2014), we included another experimental condition which has a similar canonical SVO structure as (5a), but a gender-mismatching pronoun ‘she’ (ta_female_/她) rather than the matching form ‘he’ (tamale/他) was used as the critical word in the second clause (See 6d in **Table 1**).

**Table 1:** Experimental conditions and exemplar sentences for Experiment 1 with approximate literal translations.

Conditions	Examples
NP1_sub_reference (SVO)	(6a). 爷爷读过了早报, 他/今天/起得/特别早.


	* Yeye duguole zaobao, ta_male_/jintian/qide/tebiezao*
	* Grandpa read Morning newspaper, ***he/*** get up/ very early/ today*.
	Grandpa has read the Morning newspaper, **he/** got up/ very early/ today.
NP2_sub_ reference (OSV)	(6b). 早报爷爷读过了, 他/今天/起得/特别早.


	* Zaobao yeye duguole, ta_male_/jintian/qide/tebiezao*.
	* Morning newspaper Grandpa read*, ***he/*** *get up/ very early/ today*.
	Grandpa has read the Morning newspaper, **he/** got up/ very early/ today.
NP1_topic_ reference (SOV)	(6c). 爷爷早报读过了, 他/今天/起得/特别早.


	* Yeye zaobao duguole, ta_male_/jintian/qide/tebiezao*.
	* Grandpa Morning newspaper read*, ***he/*** *get up/ very early/ today*.
	Grandpa has read the Morning newspaper, **he/** got up/ very early/today.
Gender-mismatching reference (SVO)	(6d). 爷爷读过了早报, 她/今天/起得/特别早.


	* Yeye duguole zaobao, ta_female_/jintian/qide/tebiezao*.
	* Grandpa read Morning newspaper*, ***she/*** *get up/ very early/ today.*
	Grandpa has read the Morning newspaper, **she/** got up/ very early/ today.

Based on previous ERP studies of pronoun resolution (Qiu et al., 2012; Xu et al., 2013; Xu and Zhou, under revision) and psycholinguistic theories of anaphora resolution (Garrod and Sanford, 1994; Garrod and Terras, 2000), we predicted that the neural resources devoted to resolving the pronoun toward a prominent antecedent should be different from those toward a less prominent antecedent. Specifically, we predicted that if pragmatically encoded information status (e.g., aboutness, givenness) can increase the salience of a referent, a pronoun referring to such a referent (e.g., *Grandpa* in 5c) should elicit a less positive P600 response than the referent which does not carry such features (e.g., *Grandpa* in 5a) when the other structural differences (including both surface structure such as order of mention and deep structure such as grammatical role e.g., subject or object) were held constant across conditions. However, if the surface/linear constraints such as order of mention dominate pronoun interpretation, we predicted that the pronouns referring to referents in both the SVO structure (5a) and the SOV structure (5c) should elicit similar ERP responses, and that both would be smaller than the ERPs elicited by the pronouns in the OSV structure (5b), since the referents are linearly more prominent in the former than the latter cases. Moreover, according to previous relevant studies (Osterhout and Mobley, 1995; Xu et al., 2013; Nieuwland, 2014), relative to each gender-matching condition, the pronouns in the gender-mismatching condition should elicit enhanced late positivities (P600s).

### Methods

#### Participants

Twenty-eight native Chinese speakers from Nanjing Normal University, Nanjing, China (18 females, age ranging from 18 to 28 years with mean age of 24.1 years) were compensated for the participation. All of them were right-handed and had normal or corrected-to-normal vision. None of them had a history of neurological or psychiatric disorders. Subjects gave informed consent before the experiment. This study was carried out in accordance with the Declaration of Helsinki and was approved by the Ethics Committee of the Nanjing Normal University.

#### Materials

One hundred and twenty quartets of two-clause sentences were constructed (30 sentences per condition). The initial clause of each sentence has three versions, namely the SVO structure, the OSV structure, and the SOV structure, as illustrated in sentences (5a), (5b), and (5c), respectively. The second clause, which remained identical across gender-matching conditions, was initiated by a third personal pronoun (*he* or *she*) referring to the antecedent embedded in the initial clause. The combination of the two clauses formed three types of coreferential relations (See **Table 1**): the NP1_sub_ reference (i.e., SVO structure) like (6a) in which a pronoun was coreferential with the sentence-initial entity which functions as subject, the NP2_sub_ reference (OSV structure) like (6b) in which a pronoun was coreferential with the second-mentioned entity which functions as subject, and the NP1_topic_ reference (i.e., SOV structure) like (6c) in which a pronoun was coreferential with the sentence-initial entity which functions as sentence topic. In addition, there is a fourth condition (i.e., gender-mismatching reference) which has the same structure as the NP1_sub_ reference, with the exception that the gender information of the pronoun mismatched its antecedent (see 6d). Although the animate noun in the initial clause (e.g., *Grandpa*) plays the same thematic role (i.e., agent), the information status carried by the animate noun was different across conditions: it functions purely as the grammatical subject in NP1_sub_ reference and NP2_sub_ reference, but additionally encodes pragmatically based information (e.g., aboutness, givenness) in NP1_topic_ reference. Moreover, although the animate noun plays a similar syntactic role in NP1_sub_ reference and NP2_sub_ reference, the information status carried by the animate referent was not identical either, since it was first-mentioned in the former but second-mentioned in the latter cases. These differences may modulate the accessibility status of the referents and thereby affect the neural responses underlying pronoun interpretation.

In order to examine to what extent each of the experimental sentences was acceptable, an off-line sentence acceptability rating test was conducted prior to the ERP experiment. For this test, the critical sentences, together with filler sentences, were divided into four versions using a Latin-square procedure. Twenty students were randomly assigned to one of the four versions and were asked to judge the acceptability of each of the sentences using a 7-point Likert Scale (1 indicating the least acceptable and 7 indicating the most acceptable). Results (See **Table 2**) showed that the NP1_sub_ reference (SVO sentence) had higher acceptability than either the NP1_topic_ reference (SOV sentence) or the NP2_sub_ reference (OSV sentence), *p*s < 0.001, while there was no significant difference between the NP1_topic_ reference and the NP2_sub_ reference (*p* > 0.05). This is consistent with the linguistic argument that the SVO is a canonical structure in Chinese whereas the SOV structure or the OSV structure, as the derived form from the SVO structure, is considered to be non-canonical (Li and Thompson, 1976; LaPolla, 1995; Huang et al., 2009). Moreover, the gender-mismatching reference was rated much less acceptable than each of other three types of gender-matching references (*p*s < 0.01).

**Table 2:** Results from the acceptability test.

	Acceptability test	
	Mean	SD
NP1_sub_ reference (SVO)	5.91	0.62
NP2_sub_ reference (OSV)	4.32	1.53
NP1_topic_ reference (SOV)	4.11	1.36
gender-mismatching reference (SVO)	2.51	1.13

In the ERP experiment, each critical sentence in a quartet was assigned to a different testing list with a Latin-square procedure, such that in each list there were 30 sentences per condition. One hundred and twenty filler sentences were added to each list. In order to encourage readers to read the sentence naturally and avoid adopting certain processing strategies, the fillers were composed of various two-clause sentence constructions (half containing pronouns (e.g., *he*, *she*, and *they*) and the other half without), with an SVO structure for the initial clause. Sentences in each list were then pseudo-randomized, with the restriction that no more than three consecutive sentences were of the same condition. Equal numbers of participants were randomly assigned to each of the four lists.

### Procedures

Participants sat in a comfortable chair in a dimly lit room and were instructed to read each sentence attentively. Each trial began with a fixation point (“+”) at the center of the screen for 500 ms, followed by a blank screen for 500 ms. Then the initial clause was presented as a whole on the screen. After reading the initial clause, the participant immediately pressed the space bar to initiate the second clause (the clause began with the critical pronoun *he* or *she*), which was presented segment-by-segment at the center of the screen. Each segment was presented for 400 ms, followed by a blank screen for another 400 ms. The final segment of each sentence was followed by a yes/no comprehension question that probed knowledge related to the whole sentence. The assignment of hand to response type was counterbalanced across participants.

The participant performed a practice block of 16 sentences, which had similar structures as the test stimuli in the formal experiment. The test stimuli were divided into four blocks, with breaks of about 4 min between each block. The test of each participant lasted about 2 h, including electrode preparation.

### EEG Recording and Data Analysis

EEG activity was recorded from 64 electrodes in a secured elastic cap (Electro-cap International). The EEGs were referenced online to the tip of nose and re-referenced oﬄine to the algebraic average activity measured in the left and right mastoids (TP9 and TP10). The vertical electrooculogram (VEOG) was monitored from an electrode located above the right eye and the horizontal electrooculogram (HEOG) from an electrode located at the outer canthus of the left eye. Electrode impedances were kept below 5 kΩ. EEG signals were filtered using a bandpass of 0.016–70 Hz, and digitized at a sampling rate of 500 Hz.

For each critical sentence, a 1000 ms ERP epoch was extracted for the critical pronoun in the second clause, with a 200 ms pre-stimulus baseline and the ERP response to the pronoun for 800 ms. Trials with incorrect responses in the comprehension task, or with EEG maximum amplitude exceeding ±60 μV, were eliminated from data analysis, and the remainder were screened for drift artifacts. The mean number of trials included for EEG analysis was 25.7 for the SVO reference condition, 25.8 for the OSV reference condition, 26 for the SOV reference condition, and 25.5 for the gender-mismatching reference condition, which did not differ between conditions. On the basis of visual inspection as well as the relevant literatures (Li and Zhou, 2010; Qiu et al., 2012; Xu et al., 2013), the 200–300 ms window was selected to capture early ERP components, and the 300–500 ms and 500–800 ms time windows were selected for statistical analysis of the P600s.

First, omnibus repeated-measures analyses of variance (ANOVA) were conducted on mean ERP amplitudes in each of the three time windows, with *experimental condition* (four levels: NP1_sub_ reference, NP2_sub_ reference, NP1_topic_ reference, and gender-mismatching reference), *region* (three levels: frontal vs. central vs. posterior), and *hemisphere* (three levels: left vs. middle vs. right) as within-participant variables. These were followed by two separate ANOVAs in the midline and the lateral regions, respectively. The interactions between *experimental condition* and *region* (and/or *hemisphere*) were followed up by separate ANOVAs examining the effect of *experimental condition* in each region or hemisphere. Significant main or simple effects of *experimental condition* were followed up by carrying out pair-wise comparisons comparing each type of experimental condition with one another.

For the midline analysis, there were three regions of interest: frontal (Fz and FCz), central (Cz and CPz), and posterior (Pz and POz). For the lateral analysis, the region and hemisphere were crossed, resulting in six regions of interest: left frontal (F1, F3, F5, FC1, FC3, and FC5), left central (C1, C3, C5, CP1, CP3, and CP5), left posterior (P1, P3, P5, PO3, and PO7), right frontal (F2, F4, F6, FC2, FC4, and FC6), right central (C2, C4, C6, CP2, CP4, and CP6), and right posterior (P2, P4, P6, PO4, and PO8). Mean amplitudes over electrodes in each region of interest were entered into ANOVAs. Bonferroni correction was used for multiple comparisons. The Greenhouse–Geisser correction was applied when appropriate (Geisser and Greenhouse, 1959).

### Results

#### Behavioral Results

The average comprehension accuracy was 97.5% (Mean = 29.3, SD = 0.80) for the OSV reference condition, 98.3% (Mean = 29.5, SD = 0.96) for the SOV reference condition, 98.0% (Mean = 29.4, SD = 0.83) for the SVO reference condition, and 97.3% (Mean = 29.2, SD = 1.09) for the gender-mismatching reference condition. The average comprehension accuracy was not significantly different between conditions, suggesting that those sentences are equally easy to understand.

#### ERP Results

**FIGURE 2:**
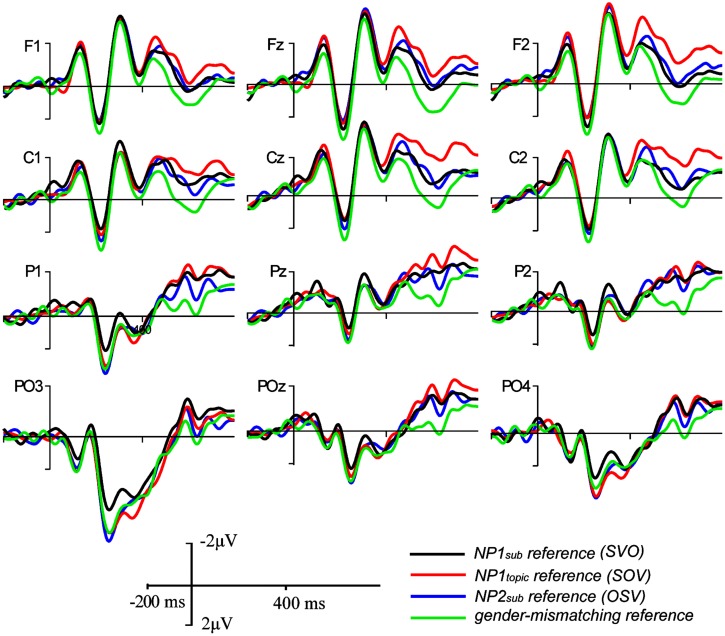
**Grand average ERPs time-locked to the critical pronoun in Experiment 1 for the NP1_sub_ reference (SVO structure), the NP1_topic_ reference (SOV structure), the NP2_sub_ reference (OSV structure), and the gender-mismatching reference conditions, respectively**.

**FIGURE 3:**
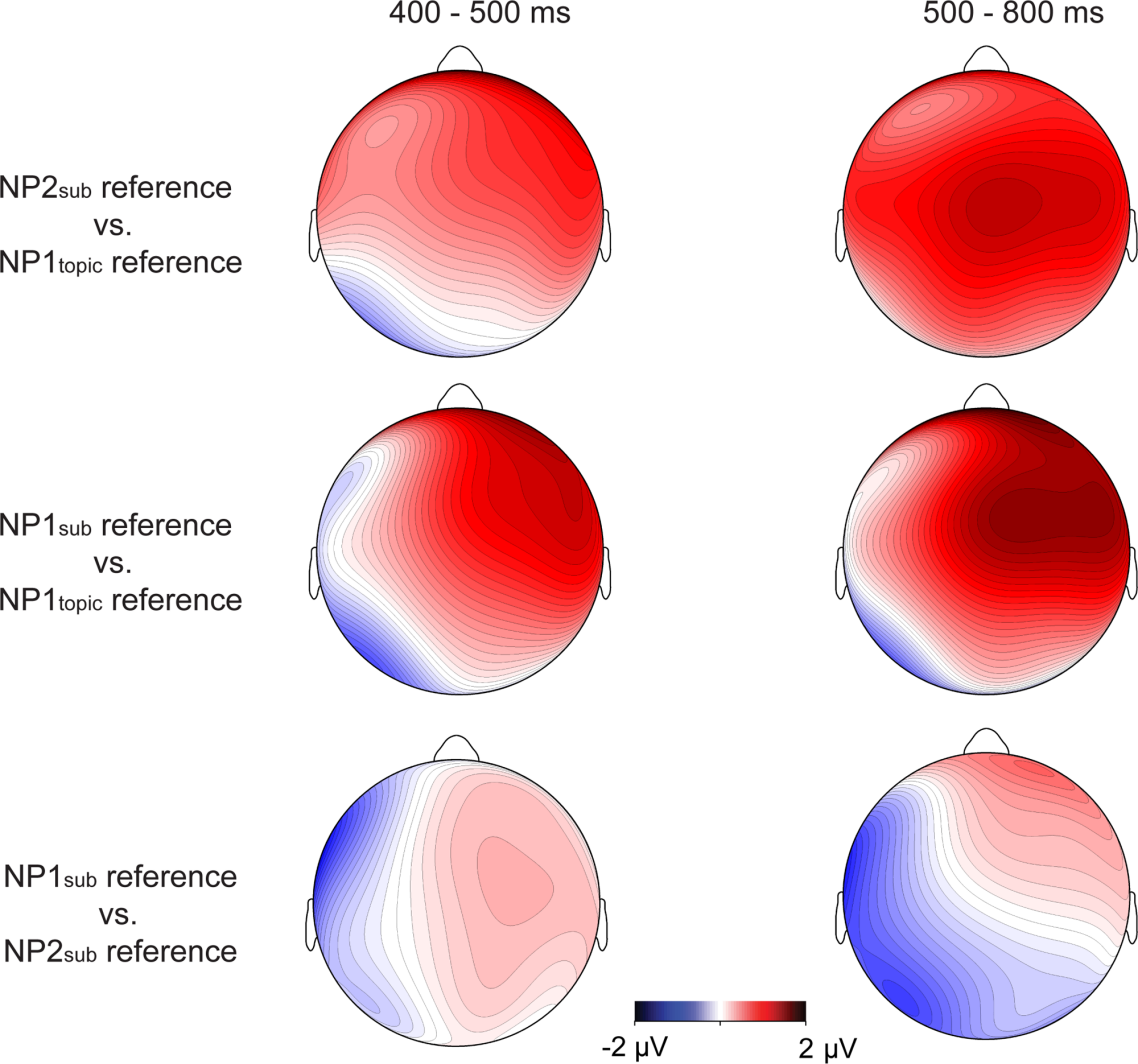
**Topographic maps for difference waves on the pronoun in Experiment 1 between the NP2_sub_ reference and the NP1_topic_ reference, and between the NP1_sub_ reference and the NP1_topic_ reference, and between the NP1_sub_ reference and the NP2_sub_ reference in 400–500 ms window (left column) and 500–800 ms window (right column), respectively**.

**FIGURE 4:**
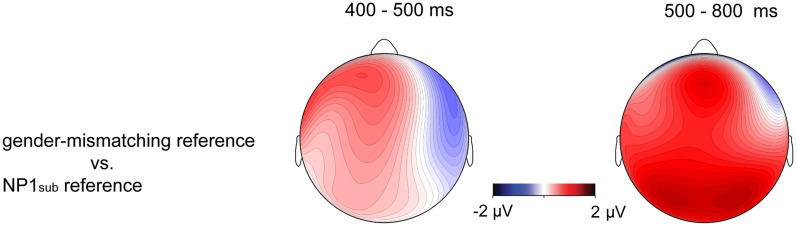
**Topographic maps for difference waves on the pronoun in Experiment 1 between the NP1_sub_ reference (gender-matching reference with SVO structure) and the gender-mismatching reference (with SVO structure) in 400–500 ms window (left) and 500–800 ms window (right), respectively**.

The grand-average ERP waveforms and the topographic maps for difference waves, as shown in **Figures 2 and 3**, showed that larger late positive ERP responses were evoked by the pronouns in the NP1_sub_ reference and the NP2_sub_ reference compared to the pronouns in the NP2_topic_ reference, whereas there was no clear difference in P600 responses between the NP1_sub_ reference and the NP2_sub_ reference. Moreover, compared with the gender-matching reference conditions, the pronouns in the gender-mismatching reference condition elicited larger later positive ERP responses (see **Figures 2 and 4**). Statistical analyses confirmed these observations. The omnibus ANOVA with *experimental condition*, *region*, and *hemisphere* as within-subject variables consistently showed a significant interaction between *experimental condition* and *hemisphere* (*p*s < 0.05) across all time windows (200–300 ms, 300–500/400–500 ms, and 500–800 ms). In the following, we only reported the results of the two separate ANOVAs from the lateral and the midline analyses, respectively.

#### 200–300 ms

Repeated-measures ANOVA with *experimental condition*, *region*, and *hemisphere* (in the lateral analysis) as within-participant factors showed a significant interaction between *condition* and *region F*(6,162) = 5.69, *p* < 0.01, although there was no main effect of *condition F*(3,81) = 1.44, *p* > 0.2. Further analysis to resolve the interaction showed that relative to pronouns in NP1_sub_ reference, larger positive responses were evoked by pronouns in NP2_sub_ reference *F*(1,27) = 8.09, *p* < 0.01, NP1_topic_ reference *F*(1,27) = 6.72, *p* < 0.02, and gender-mismatching reference *F*(1,27) = 15.58, *p* < 0.005 in the bilateral posterior areas. No effects of interest were found in the midline analysis.

#### 300–500 ms

In the lateral analysis, repeated-measures ANOVA showed a significant interaction between *condition* and *hemisphere F*(3,81) = 2.87, *p* < 0.05, and between *condition* and *region F*(6,162) = 2.97, *p* < 0.05. No other main effects or interactions of interest were significant. Further analysis to resolve the interaction between *condition* and *region* showed that pronouns in NP1_topic_ reference evoked less positive activities than pronouns in gender-mismatching reference in frontal regions (F1/2, F3/4, F5/6, FC1/2, FC3/4, and FC5/6), *F*(1,27) = 3.32, 0.05 < *p* < 0.1. The interaction between *condition* and *hemisphere* showed that pronouns in NP1_sub_ reference evoked less positive activities than pronouns in the gender-mismatching reference *F*(1,27) = 2.93, 0.05 < *p* < 0.1 in the left hemisphere. No other effects were significant. In the midline, neither the main effects nor the interactions were significant.

#### 400–500 ms

The absence of the modulation of experimental manipulation in the 300–500 ms time window may be due to the wide time window for measuring the early P600 effect. To further explore this possibility, a smaller time window, namely 400–500 ms, was additionally analyzed.

In the lateral analysis, repeated-measures ANOVA showed a significant interaction between *condition* and *region F*(6,162) = 2.50, *p* < 0.05. Further analysis to resolve this interaction showed that relative to NP1_topic_ reference, larger positive responses were evoked by pronouns in NP1_sub_ reference *F*(1,27) = 3.12, 0.05 < *p* < 0.1, NP2_sub_ reference *F*(1,27) = 4.65, *p* < 0.05, and gender-mismatching reference *F*(1,27) = 4.52, *p* < 0.05 in frontal regions (F1/2, F3/4, F5/6, FC1/2, FC3/4, and FC5/6), although no effects of interest were found in central or posterior regions. No significant effects relevant to the experimental manipulation were found in the midline analysis.

#### 500–800 ms

In the lateral analysis, repeated-measures ANOVA revealed a significant main effect of *condition F*(3,81) = 3.71, *p* < 0.02, and significant two-way interactions between *condition* and *hemisphere F*(3,81), = 4.55, *p* < 0.01, and between *condition* and *region* (marginal significant) *F*(6,162) = 2.28, 0.05 < *p* < 0.1.

Further analyses were performed to resolve the interaction of *condition* and *hemisphere*. In the right hemisphere, larger P600 responses were elicited by pronouns in NP1_sub_ reference *F*(1,27) = 5.03, *p* < 0.05, and NP2_sub_ reference *F*(1,27) = 3.69, 0.05 < *p* < 0.1 compared to pronouns in NP1_topic_ reference. Pronouns in gender-mismatching reference evoked larger positive deflections than pronouns in NP1_topic_ reference *F*(1,27) = 13.88, *p* < 0.001, and those in NP1_sub_ reference *F*(1,27) = 3.19, 0.05 < *p* < 0.1. In the left hemisphere, larger P600 responses were elicited by pronouns in gender-mismatching reference compared to pronouns in NP1_topic_ reference *F*(1,27) = 6.72, *p* < 0.02, and those in NP1_sub_ reference *F*(1,27) = 3.92, *p* < 0.06. No other comparisons were significant in the left hemisphere. Further analyses to resolve the interaction between *condition* and *region* showed that relative to NP1_topic_ reference, larger P600 responses were elicited by pronouns in NP1_sub_ reference *F*(1,27) = 5.18, *p* < 0.04, NP2_sub_ reference *F*(1,27) = 4.66, *p* < 0.05, and gender-mismatching reference *F*(1,27) = 7.58, *p* < 0.01 in frontal electrodes (F1/2, F3/4, F5/6, FC1/2, FC3/4, and FC5/6). Similar pattern of results were observed in central electrodes (C1/2, C3/4, C5/6, CP1/2, CP3/4, and CP5/6) although the effect was smaller—that is, larger P600 responses were elicited by pronouns in NP1_sub_ reference *F*(1,27) = 3.16, 0.05 < *p* < 0.1, and NP2_sub_ reference *F*(1,27) = 4.83, *p* < 0.04 relative to pronouns in NP1_topic_ reference. Moreover, larger P600 responses were evoked by pronouns in gender-mismatching reference than pronouns in NP1_topic_ reference *F*(1,27) = 14.75, *p* < 0.001 and NP1_sub_ reference *F*(1,27) = 3.6, *p* < 0.07 in the central regions. There was no significant difference in P600 amplitude between NP2_sub_ reference and NP1_sub_ reference in each region or hemisphere, *p*s > 0.1.

In the midline analyses, repeated-measures ANOVA revealed a significant main effect of *condition F*(3,81) = 6.38, *p* < 0.002. Pairwise comparisons showed that larger P600 responses were elicited by pronouns in NP1_sub_ reference *F*(1,27) = 5.16, *p* < 0.04 and NP2_sub_ reference *F*(1,27) = 5.28, *p* < 0.04, relative to NP1_topic_ reference. Moreover, larger P600 responses were elicited by pronouns in gender-mismatching reference relative to pronouns in NP1_topic_ reference *F*(1,27) = 22.43, *p* < 0.001, NP1_sub_ reference *F*(1,27) = 4.88, *p* < 0.04, and NP2_sub_ reference *F*(1,27) = 4.84, *p* < 0.04. Again, there was no difference between NP1_sub_ reference and NP2_sub_ reference *F* < 1.

### Discussion

In summary, in the 200–300 ms time window, there was no significant difference between conditions in the frontal–central regions, although a reduced positive response was elicited by the pronoun in the NP1_sub_ reference condition compared to each of the other conditions in the posterior region. Moreover, although there was no effect of information status in the 300–500 ms time window, the effect of information status was apparent in the 400–500 ms and 500–800 ms: enhanced positive responses were elicited by pronouns in the NP1_sub_ reference condition and the NP2_sub_ reference condition relative to pronouns in the NP1_topic_ reference condition but there was no difference between the pronouns in the NP1_sub_ reference and NP2_sub_ reference conditions. In addition, larger positive responses were elicited by pronouns in the gender-mismatching reference condition relative to pronouns in each of the other gender-matching reference conditions in both 300–500 (or 400–500) and 500–800 ms time windows. These findings suggest that pragmatically encoded topichood can increase the salience of the referent and thereby facilitate the interpretation of the pronoun, whereas purely structural-based order of mention has only limited impact upon pronoun interpretation.

Although similar positive effects (P600s) were elicited by the gender-mismatching condition and the information structure manipulation conditions, they are different in topographic distribution. As addressed previously, P600 is not one effect but a family of effects, its topographical differences may correspond to different functional interpretations (Hagoort et al., 1999; Molinaro et al., 2008b, 2011a,b). Note that the P600 effects evoked by gender-matching pronouns in the present experiment were frontally distributed and dissociable from the central-posteriorly distributed P600 as revealed by a number of previous studies concerning syntactic/agreement violations (e.g., Osterhout and Mobley, 1995; Osterhout et al., 1997; Molinaro et al., 2008a). It has been argued that the processing costs associated with overwriting the preferred or the most active structural representation of a sentence, like the unpreferred continuations in garden-path sentences, result in a more frontally distributed P600, whereas a collapse of the structural representation as in outright syntactic or agreement violations (e.g., gender, number) generally results in a more posteriorly distributed P600 (Hagoort et al., 1999; Kaan and Swaab, 2003; Molinaro et al., 2008a,b). Consistent with this interpretation, a stronger P600 effect elicited by non-topic referring pronouns (e.g., NP1_sub_ reference/NP2_sub_ reference) relative to topic-referring pronouns (e.g., NP1_topic_ reference) in this experiment was frontally distributed (see **Figure 3**), probably due to the absence of syntactic incongruence in the critical sentences. Rather, the P600 effects were mainly related to the differences in terms of information status regarding the referents: a pronoun is preferentially interpreted as coreferential with a more accessible entity (e.g., topic) rather than a less accessible one (e.g., subject). The size of the P600 effect has been interpreted as reflecting the difficulty of integrating the pronoun-antecedent relation as well as the discourse context into a coherent discourse representation (Callahan, 2008; Silva-Pereyra et al., 2012; Xu et al., 2013). In contrast, the P600 effect evoked by a pronoun with gender incongruence was centro-posteriorly and broadly distributed, as illustrated in **Figure 4** (especially in the late time window), a finding which has been reported by a number of previous studies concerning pronoun resolution (Osterhout and Mobley, 1995; Osterhout et al., 1997; Silva-Pereyra et al., 2012; Xu et al., 2013). The P600 in the latter case was interpreted as reflecting the recomputation of the referential representation after a conflict has been detected. In particular, the P600 effect for the gender-mismatching pronoun here is more widely distributed, as illustrated in **Figure 4**, presumably because the integration of a gender-mismatching pronoun into discourse representation requires additional processing resources. Nonetheless, it indicates that the neural processes underlying processing the information-inappropriate reference are dissociable from processing the grammar-incongruent reference.

The failure to observe any clear negative modulations in the early time window (200–300 ms) is inconsistent with previous studies concerning referential processing in Romance languages (e.g., Spanish, Dutch; Demestre et al., 1999; Lamers et al., 2006, 2008) in which the morphological incongruence (e.g., syntactic gender, case) between pronoun/null pronoun and the antecedent regularly generated early negativities, reflecting the initial establishment of a co-referential relation based mainly on morpho-syntactic rules (e.g., gender and number agreement). However, this is consistent with a number of previous studies concerning pronoun resolution in Chinese (Xu et al., 2013; Xu and Zhou, under revision) and English (Osterhout and Mobley, 1995; Osterhout et al., 1997) in which only a monophasic positivity was found. The absence of such early negative modulation in the later case was presumably because the coreferential relation was not initially computed on the basis of form rules, given the lack of morphological markers to signal the pronoun-antecedent relation in both Chinese and English.

However, although there were no early ERP effects in the frontal-central areas, NP1_sub_ reference evoked reduced positivity compared to the other conditions in the posterior areas (see **Figure 2**). Previous studies concerning visual attention reported larger posterior positivities for stimuli occurring less frequently (Donchin, 1984; Hillyard, 1984) because of the increased attentional engagement for processing these unfamiliar events. As for language processing, Heine et al. (2006) found that a pronoun referring to a less frequent antecedent elicited a larger positivity peaking around 300 ms post onset (i.e., P300), suggesting that increased attentional processing was required for retrieving the low-frequency antecedent. Similarly, the reduced posterior positivity in the SVO condition relative to the other conditions could also be related with the different attentional involvement for retrieving the antecedent from these different constructions, given that the canonical SVO structure happens more frequently than the other non-canonical structures (e.g., SOV, OSV), as suggested by the higher acceptability score in the former than the latter cases.

## Experiment 2

Results from Experiment 1 suggest that topic status can increase the salience of an antecedent during reference processing, with larger P600s for non-topic referring pronouns than topic-referring pronouns, whereas order of mention has no clear contribution to P600 amplitude. However, one might argue that the failure to find the effect of order of mention on P600 amplitude may be attributed to the relatively highly accessible status of the antecedent entity in each condition, since only one unambiguous animate referent was available for the pronoun in Experiment 1. The occurrence of an inanimate entity in object position cannot generate effective competition with the animate entity in subject position during on-line pronoun interpretation. To examine whether the absence of the order of mention effect was due to the lack of referential competition, Experiment 2 used stimuli that included two animate entities, which would potentially increase the competition during pronoun interpretation.

Moreover, it is plausible that the observed reduced P600 for NP1_topic_ reference relative to NP1_sub_ reference in Experiment 1 is related to the lower acceptability of the SOV compared to the SVO sentences, as indicated by the pretest, rather than to the higher prominence of the topic entity than the subject entity. One potential reason for the lower acceptability of SOV sentences may be the lack of explicit topic devices conveying topic information. Although Chinese is regarded as a topic-prominent language, in many cases the identification of a topic in Chinese is dependent on the marked forms such as *ne*, *a*, *ya* or just a pause particle, i.e., a comma, separating topic from comment (Li and Thompson, 1989; Xu and Liu, 2007). In particular, the use of a comma to separate topic from comment applies not only to topic-prominent languages such as Chinese but also other non-topic prominent languages. To extend the findings of Experiment 1, the topic entity in Experiment 2 not only occurs sentence initially but also is separated from the comment by a comma.

If the null effect of mention of order was due to the lack of competition when the context only had one available animate antecedent, then an order mention effect should be observed in Experiment 2 in which two animate antecedents were simultaneously presented. If, however, order of mention was independent of the competition status of the potential antecedents, the same pattern as Experiment 1 should be obtained. Moreover, if the enlarged P600 responses evoked by pronouns in NP1_sub_ reference (SVO structure) compared to pronouns in NP1_topic_ reference (SOV structure) stem mainly from the difference in off-line acceptability rating rather than the more prominent cognitive status of the former than the latter cases, there should be no difference in the P600 amplitude when the acceptability scores are comparable between the topic structure and the non-topic structure.

### Materials

Similar to Experiment 1, three types of critical coreferential relations were constructed: (1) the pronoun is coreferential with the sentence-initial NP which functions as sentence topic (i.e., NP1_topic_ reference), (2) the pronoun is coreferential with the initial NP which functions as grammatical subject (i.e., NP1_sub_ reference), (3) the pronoun is coreferential with the second mentioned NP which functions as grammatical subject (i.e., NP2_sub_ reference). For all these critical conditions, one main difference between the two experiments is that while only one animate antecedent was used in Experiment 1, two animate antecedents were adopted in Experiment 2. Moreover, the topic structure (OSV structure) in Experiment 2 was markedly separated from the comment by a pause particle, i.e., a comma (see 7b/c in **Table 3**). Additionally, it should be noted that different from the one-animate situation where the initial noun plays an agent role (in both SVO and SOV), when the topic structure contains two animate antecedents; the initial animate noun (topic entity) is regularly interpreted as playing a patient role (a topicalized object). Instead, the second animate noun plays an agent role and thus is the subject of the sentence (see 7b/c). For this reason, we only adopted the SVO structure (7a) and OSV structure (7b/c) in Experiment 2. Ninety triplets of critical sentences were constructed, with 30 sentences for each condition. Each sentence contained two animate antecedents with different gender features denoted by proper names (e.g., 小明/*Xiaoming*, a typical male name, and 小兰/*Xiaolan*, a typical female name). For half of the sentences, NP1 was male and NP2 female, whereas for the other half, NP1 was female and NP2 male. Each critical sentence in a triplet was assigned to a different testing list with a Latin-square procedure, such that each participant had equal chance to encounter the pronoun *he* and *she* in each list. In addition to the critical sentences, 150 filler sentences with various structures were constructed, including 30 sentences with two ambiguous antecedents (one pronoun with two potential gender-matching animate antecedents), 90 sentences with one animate antecedent, and 30 sentences with no animate antecedent.

**Table 3:** Experimental conditions and exemplar sentences for Experiment 2 with approximate literal translations.

Conditions	Examples
NP1_sub_ reference (SVO)	(7a). 小明认识小兰, 他/经常/去/那家/咖啡厅.


	* Xiaoming_male_ renshi Xiaolan_female_*,***ta_male_*** *jingchang qu najia kafeiting*.
	* Xiaoming know Xiaolan*,***he****/ always/ go to/ that/ coffee shop.*
	Xiaoming knows Xiaolan. **He** (Xiaoming) always goes to that coffee shop.
NP1_topic_ reference (OSV)	(7b). 小兰, 小明认识, 她/经常/去/那家/咖啡厅.


	* Xiaolan_female_, Xiaoming_male_ renshi*, ***ta_female_****/ jingchang/ qu/ najia/ kafeiting*.
	* Xiaolan, Xiaoming know*, ***she****/ always/ go to/ that/ coffee shop.*
	Xiaolan, Xiaoming knows. **She** (Xiaolan) always goes to that coffee shop.
NP2_sub_ reference (OSV)	(7c). 小兰, 小明认识, 他/经常/去/那家/咖啡厅.


	* Xiaolan_female_, Xiaoming_male_ renshi*, ***ta_male_*** *jingchang qu najia kafeiting*.
	* Xiaolan, Xiaoming know*, ***he****/ always/ go to/ that/ coffee shop.*
	Xiaolan, Xiaoming knows. **He** (Xiaoming) always goes to that coffee shop.

Each critical sentence in a triplet was assigned to a different test list with a Latin square procedure, such that in each list there were 30 sentences per experimental condition and each typical name had equal chance to appear in the initial (i.e., NP1) or second (i.e., NP2) position. The procedures to assign stimuli into the test lists were the same as Experiment 1.

As in Experiment 1, an acceptability rating was carried out with another twenty students who did not participate in the ERP studies. They were randomly assigned to one of the three versions and were asked to judge the acceptability of each sentence using a 7-point Likert Scale (1 indicating the least acceptable and 7 indicating the most acceptable). As demonstrated in **Table 4**, both NP1_topic_ reference and NP1_sub_ reference sentences were more acceptable than NP2_sub_ reference sentence (*p*s < 0.01), but the difference between NP1_topic_ reference and NP1_sub_ reference sentences was not significant (*p* > 0.1).

**Table 4:** Results from the acceptability test.

	Acceptability test	
	Mean	SD
NP1_sub_ reference (SVO)	5.08	0.92
NP2_sub_ reference (OSV)	3.75	1.21
NP1_topic_ reference (OSV)	4.62	1.10

### Participants

Twenty-four native Chinese speakers (17 females, age ranging from 20 to 26 years with mean age of 22.9 years), who did not take part in Experiment 1, were recruited from Nanjing Normal University. All of them were right-handed and had normal or corrected-to-normal vision. None of them had a history of neurological or psychiatric disorder. Subjects gave informed consent before the experiment.

### EEG recording and data analysis

The procedures including presenting stimulus, collecting and analyzing the EEG data were the same as in Experiment 1. The mean number of trials included for EEG analysis was 27.2 for the NP1_topic_ reference, 27.8 for the NP2_sub_ reference, and 27.7 for the NP1_sub_ reference, with no significant difference between conditions (*p*s > 0.1).

### Results

#### Behavioral Results

The average comprehension accuracy was 95% (Mean = 28.50, SD = 1.14) for the NP1_topic_ reference, 96.8% (Mean = 29.04, SD = 1.08) for the NP2_sub_ reference, and 97.8% (Mean = 29.33, SD = 0.92) for the NP1_sub_ reference. Paired-t tests showed that the average comprehension accuracy was higher for NP1_sub_ reference than for the NP1_topic_ reference, *t*(1,23) = 17.97, *p* < 0.01, and that no other comparisons were significant.

#### EEG Results

Similar to Experiment 1, given that the omnibus ANOVA with *experimental condition*, *region*, and *hemisphere* as within-subject variables consistently showed a significant interaction between *experimental condition* and *hemisphere* (*p*s < 0.05) across all time windows, we report separate lateral and midline analyses.

#### 200–300 ms

Repeated-measures ANOVA with *experimental condition*, *region* and *hemisphere* as within-participant factors showed a significant interaction between *condition* and *hemisphere* in the lateral analysis, *F*(2,46) = 7.88, *p* < 0.01. However, the following analyses to resolve the interaction failed to show a significant effect of *condition* in either the left hemisphere *F*(2,46) = 1.51, *p* > 0.2, or the right hemisphere *F*(2,46) = 1.40, *p* > 0.2. No significant effects relevant to the experimental manipulation were found in the midline.

#### 300–500 ms

Repeated-measures ANOVA showed a significant two-way interaction between *condition* and *hemisphere F*(2, 46) = 9.17, *p* < 0.01 and a marginal three-way interaction between *condition*, *region*, and *hemisphere*, *F*(4,92) = 1.97, 0.05 < *p* < 0.1. No other main effects or interactions of interest were significant.

**FIGURE 5:**
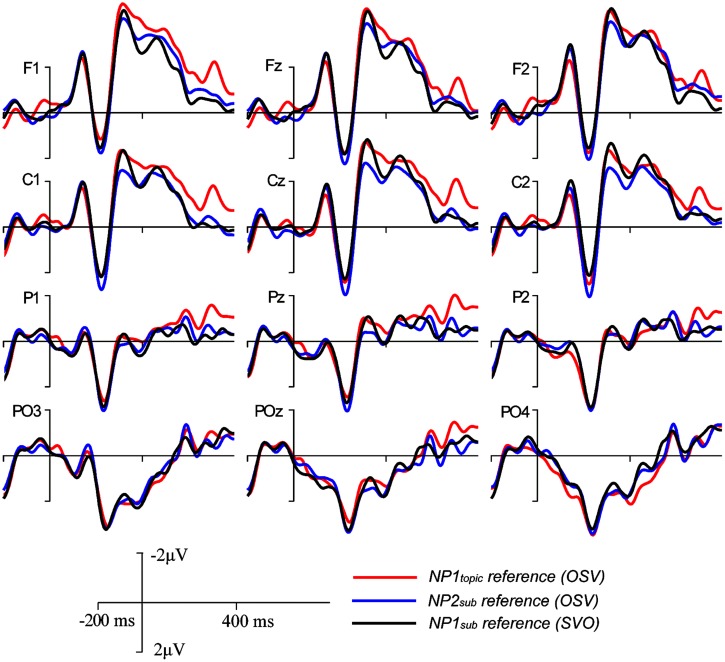
**Grand average ERPs time-locked to the critical pronoun in Experiment 2 for the NP1_topic_ reference (OSV structure), the NP2_sub_ reference (OSV structure), and the NP1_sub_ reference (SVO structure), respectively**.

**FIGURE 6:**
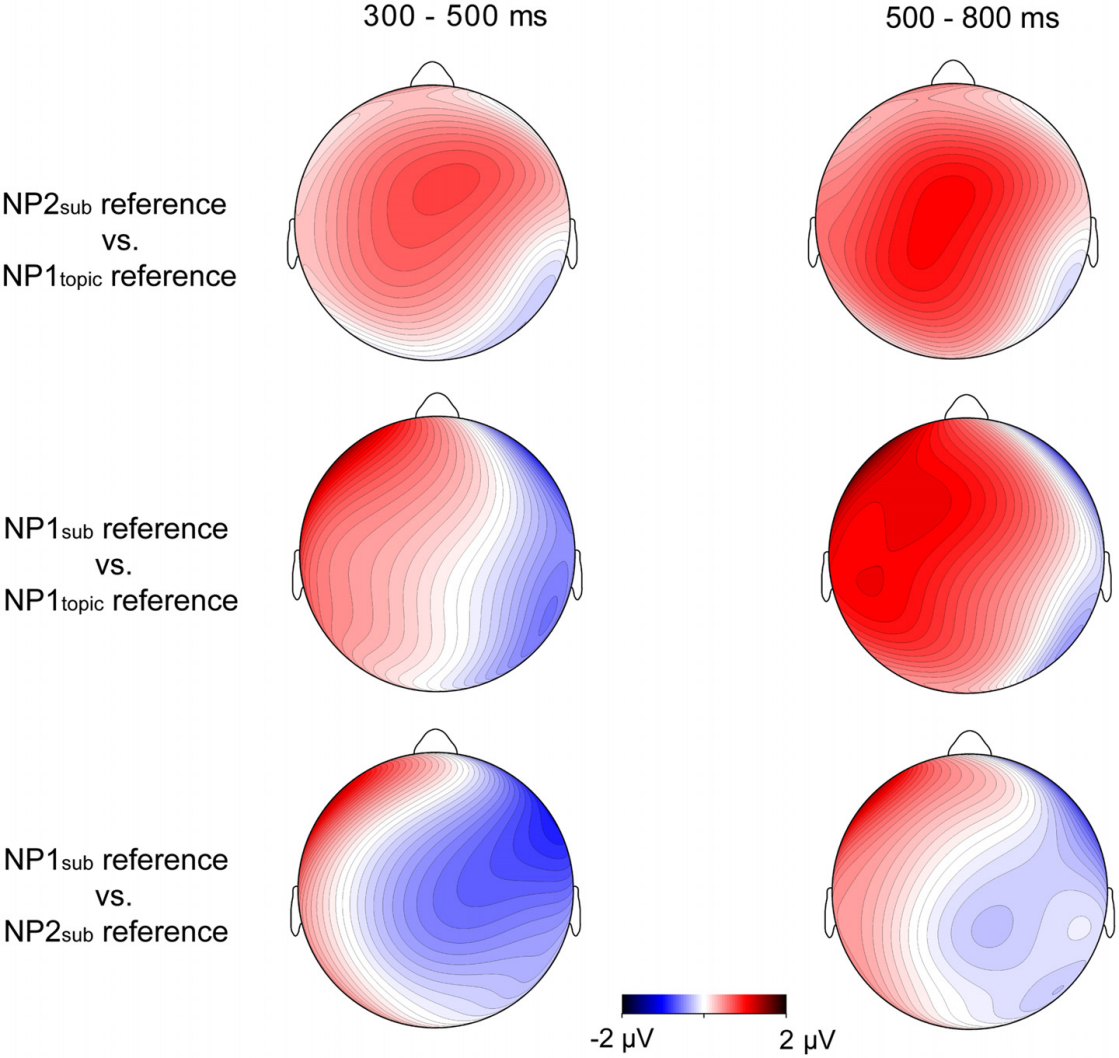
**Topographic maps for difference waves on the pronoun in Experiment 2 between the NP2_sub_ reference and the NP1_topic_ reference, and between the NP1_sub_ reference and the NP1_topic_ reference, and between the NP1_sub_ reference and the NP2_sub_ reference in 300–500 ms window (left) and 500–800 ms window (right), respectively**.

Separate analyses were carried out to resolve the three-way interaction. In the left hemisphere, relative to pronouns in NP1_topic_ reference, larger positive responses were evoked by pronouns in NP1_sub_ reference *F*(1,23) = 5.04, *p* < 0.05 (frontal region: F1, F3, F5, FC1, FC3, and FC5) and NP2_sub_ reference *F*(1,23) = 2.75, 0.05 < *p* < 0.1 (central region: C1, C3, C5, CP1, CP3, and CP5), see **Figures 5 and 6**. In the right hemisphere, however, there was no significant difference between conditions. No effects of interest were found in the midline analysis.

#### 500–800 ms

Repeated-measures ANOVA revealed a significant two-way interaction between *condition* and *hemisphere F*(2, 46) = 8.80, *p* < 0.01, although the main effect of *condition* was not significant *F*(2,46) = 2.0, *p* > 0.1. Planned comparisons were carried out to test the differences between each pair of conditions. In the left hemisphere, smaller P600 responses were elicited by pronouns in NP1_topic_ reference compared to pronouns in NP2_sub_ reference *F*(1,23) = 6.76, *p* < 0.02, and those in NP1_sub_ reference *F*(1,23) = 7.39, *p* < 0.02 (see **Figures 5** and **6**). There was no significant difference between NP2_sub_ reference and NP1_sub_ reference *F* < 1. The pairwise comparisons in the right hemisphere, however, failed to show significant differences between conditions.

Repeated-measures ANOVA in the midline analysis revealed a marginally significant effect of *condition F*(2,46) = 2.34, 0.05 < *p* < 0.1. Planned comparisons showed a similar pattern of results as in the lateral analysis, that is, smaller P600 responses were elicited by the pronouns in NP1_topic_ reference compared to the pronouns in NP2_sub_ reference *F*(1,23) = 5.82, *p* < 0.03, and to NP1_sub_ reference *F*(1,23) = 2.41, 0.05 < *p* < 0.1. Again, there was no significant difference between NP2_sub_ reference and NP1_sub_ reference *F* < 1.

### Discussion

In this experiment, we manipulated topic status by using a topic marker, i.e., a comma. We also increased the competition between antecedents by including two animate antecedents. A similar pattern of results as in Experiment 1 was observed, that is, larger P600s were evoked by pronouns in the NP2_sub_ reference and NP1_sub_ reference conditions relative to pronouns in the NP1_topic_ reference condition whereas the NP2_sub_ reference condition and the NP1_sub_ reference condition did not differ in terms of the P600. Importantly, order of mention did not modulate P600 amplitude, even when two animate entities were simultaneously presented.

Unlike in Experiment 1, in the 200–300 ms time window, we did not find any significant difference across experimental conditions either frontally or posteriorly. One possible explanation for this cross-experiment variation is that the occurrence of two animate antecedents generally gave rise to increased attentional efforts for processing the potential referential ambiguity/competition. The increased attentional resources for retrieving the correct referent may have reduced the chance to find early modulations.

In the 300–500 ms time window, both NP1_sub_ reference and NP2_sub_ reference evoked larger positive responses than NP1_topic_ reference in left frontal areas. This resembles the findings of Experiment 1 (400–500 ms time window), although the effects in Experiment 1 were mainly distributed over the right- rather than the left-frontal areas. Moreover, although similar results were obtained on the amplitude of these positivities, the time point at which such modulation emerged was different: the occurrence of this effect was earlier in Experiment 2 than in Experiment 1. This was possibly due to the increased competition induced by the presence of two animate antecedents, causing the process of integrating a pronoun with a less salient antecedent to occur earlier than the integration of a pronoun with a salient antecedent. We will come back to this issue in the Section “General Discussion.”

Although the presence of two potential animate entities increased the referential competition/complexity of pronoun resolution, the results in the 500–800 ms time window were similar to what was observed in Experiment 1. In particular, like in Experiment 1, the P600 effect in Experiment 2 was frontally distributed, probably due to the fact that there was no referential failure but only referential preference (i.e., preferred referent vs. dispreferred referent). Moreover, the finding of a larger P600 effect in NP1_sub_ reference than NP1_topic_ reference remains unchanged even when these two structures were equally acceptable as demonstrated by the pretest, suggesting that the ERP effect (P600 difference) found in Experiment 1 cannot stem from the difference in acceptability.

Although this experiment replicated the main findings of Experiment 1, some cross-experiment differences should be taken into account before arriving at the General Discussion. First, unlike in Experiment 1 where the referents of pronouns acted as grammatical subject across all conditions, the referents in Experiment 2 acted as grammatical subject in the non-topic structure (both NP1_sub_ reference and NP2_sub_ reference) but as object in the topic structure (NP1_topic_ reference). Although the present study cannot completely rule out the potential influence from grammatical role, we would like to point out that if the grammatical role exerts a more dominant influence on pronoun resolution than information status, subject referred pronouns should evoke attenuated, rather than enlarged P600 responses compared to object referred pronouns, since subject entity is more salient than object entity as demonstrated by a number of previous studies (e.g., Järvikivi et al., 2005; Wang et al., 2009). But we did not observe this pattern in the present study. Second, although the P600 was frontally maximal in each experiment, it was right distributed in Experiment 1 but left distributed in Experiment 2. The current data cannot provide an explanation for this hemispheric asymmetry, although this difference may be related to the different mechanisms underlying processing ambiguous vs. unambiguous coreference. More studies are needed to further elucidate these questions.

Finally, although the presence of two animate entities would increase the referential competition of pronoun resolution compared to the situation in which there is only one animate entity, the sentences are still unambiguous since only one antecedent matches in gender with the pronoun. Thus, it is uncertain whether the same results will hold for a situation where the two animate entities have the same gender. One possibility is that relative to the unambiguous pronoun in the non-topic structure (sentences like 7a), the ambiguous pronoun in the topic structure would give rise to an Nref, a sustained anterior negativity, which is regularly generated by the referential ambiguity in non-topic structures (e.g., van Berkum et al., 1999). Alternatively, a similar pattern of results as found in Experiment 2 could be obtained, namely an attenuated P600 elicited by an ambiguous pronoun in the topic structure than an unambiguous pronoun in the non-topic structure. If this were the case, it would provide more convincing evidence supporting the argument that topic referent is more cognitively salient than non-topic referent.

Taken together, the results from Experiment 2 revealed that a topic referent is cognitively more prominent than the non-topic referents even in cases where the pronoun has more than one potential animate antecedent, and even when the topic status of the referent is overtly marked.

## General Discussion

In this study, we found that pronouns whose referent was a topic (NP1_topic_ reference) elicited smaller P600s than pronouns whose referent was a non-topic subject, no matter whether the subject referent was in the first-mentioned position (i.e., NP1_sub_ reference in the SVO sentence) or the second-mentioned position (i.e., NP2_sub_ reference in the OSV sentence). On the other hand, P600 responses elicited by first- and second-mentioned subject referents were the same. Furthermore, the attenuation of P600 for topic-referring pronouns occurred regardless of whether there was one or two animate potential antecedents (although the P600 effect evoked by the pronoun occurred somewhat earlier in the two animate than the one animate situation). These findings suggest that pragmatic status of topicality can increase the salience of a pronoun’s referent whereas order of mention has only limited impact upon pronoun resolution.

### Information Status, P600, and the Two-Stage Model of Pronoun Resolution

As discussed in the Section “Introduction,” pronoun resolution can be differentiated into a bonding stage and a resolution stage (Garrod and Sanford, 1994; Garrod and Terras, 2000; Callahan, 2008). In the bonding stage, candidate antecedents are retrieved, whereas in resolution stage, the appropriate antecedent is integrated with the pronoun and the whole discourse. The bonding stage is not consistently reflected in ERP components, although some early negative deflections (e.g., LAN) have been reported in studies concerning morphologically rich languages (Demestre et al., 1999; Lamers et al., 2006, 2008). We did not observe clear early negativities across experiments, even though some early positive responses were observed in posterior electrodes in a context where only one animate antecedent was present (Experiment 1). The present finding is in line with previous studies of pronoun resolution in morphologically impoverished languages such as English and Chinese (Osterhout and Mobley, 1995; Osterhout et al., 1997; Xu et al., 2013), where early negativities have generally not been reported.

By contrast, the resolution stage is consistently found to be related with the occurrence of P600 regardless of cross-language variations (Qiu et al., 2012; Xu et al., 2013), reflecting the efforts taken for evaluating the pronoun-antecedent link and integrating the referential relation with the whole discourse context (Friederici, 1998; Silva-Pereyra et al., 2012). As addressed previously, P600 is not one effect but a family of effects, with the P600 related to anaphora resolution showing both an early phase and a later phase. The early part of the P600 has been argued to reflect the process of evaluating the linkage between a pronoun and its potential antecedents, whereas the later part is claimed to reflect integrating the anaphora/pronoun-antecedent relation and the whole discourse (e.g., Silva-Pereyra et al., 2012). According to this interpretation, evaluating the link between a pronoun and a less prominent antecedent is more cognitively demanding than evaluating the link between a pronoun and a prominent antecedent, and thereby gives rise to enhanced early positivities. The fact that the P600 difference emerged earlier in Experiment 2 than in Experiment 1 may thus indicate that checking the linkage between a pronoun and its potential antecedents was more difficult—triggering more activity in the early part of the P600—in Experiment 2, where there were more potential referents, and that topic privilege mitigated the difficulty more in Experiment 2 than in Experiment 1.

The later part of the P600 has been interpreted as reflecting the integration of the pronoun-antecedent relation as well as the discourse context into a coherent discourse representation (Callahan, 2008; Xu et al., 2013). The more demanding the integration processing is, the larger the amplitude of the P600 would be. The reduced P600 response evoked by pronoun referring to topic referent compared to that for the non-topic referent indicates that the process of integrating a pronoun with a more prominent entity is less demanding than the process of integrating a pronoun with a less prominent one. This P600 attenuation could not have been caused by semantic or syntactic differences, given that the referents play the same thematic roles (Experiment 1) and take identical structural positions (in both experiments).

Consistent with the above interpretation, our previous study (Xu and Zhou, under revision) has revealed that reference to a non-topic elicits a larger P600 effect (relative to reference to a topic) than reference to a non-subject (relative to reference to a subject). Those results suggest that topic information is privileged, and attempting to form coreference with a non-topic is cognitively difficult. The present experiment extends these results by showing that the attenuation of the P600 due to topic privilege is replicable when controlling for the sentential position of the topic and subject antecedents. These findings provide solid evidence to support the argument that processing a topic coreferential relation is cognitively less demanding than processing a subject coreferential relation. In other words, pragmatically-encoded aboutness can increase the prominence of the referent and therefore makes the establishment of a coreference cognitively less demanding for topic than non-topic referents.

### Order of Mention and Pronoun Resolution

When the animate referents function purely as the grammatical subject (the initial NP in SVO structure and the second NP in OSV structure), order of mention of the antecedent makes no contribution to the neural responses of the pronoun in the present study, suggesting that order of mention plays only a limited role during pronoun resolution. In particular, the absence of P600 difference remained even when two animate antecedents were presented; suggesting that the null result in Experiment 1 was unlikely to be the result of lacking referential competition. Nonetheless, when the first-mentioned NP additionally functioned as the topic of the sentence, the pronoun referring to first-mentioned antecedent evoked a reduced P600 response when compared with the pronoun referring to the second-mentioned antecedent, although the linear order of the referents was unchanged, suggesting that it is the additional assignment of topic status rather than order of mention that makes the first-mentioned referent more prominent than the second-mentioned referent.

In summary, the results from present studies suggest that pragmatic-encoded constraints such as aboutness and givenness contribute more than structurally based linear order in determining the resolution of pronoun.

### Information Status, Structural Sequence, and Pronoun Resolution

The finding of a less positive P600 to pronouns in the topic reference than in the non-topic reference provides converging evidence that information status plays a key role in pronoun resolution. This is in line with a number of previous studies concerning focus structure and pronoun/anaphora resolution. For example, evidence from behavioral studies showed that focused antecedents are processed faster when they are foregrounded in an *it*-cleft structure (Cowles et al., 2007b; Foraker and McElree, 2007; Kaiser, 2011). ERP studies (e.g., Cowles et al., 2007a; Xu and Zhou, submitted) also demonstrated that if the interpretation of a nominal anaphora (Cowles et al., 2007a) or a pronoun (Xu and Zhou, submitted) is incongruent with the informational role assigned through a *wh*-question focus structure, such inappropriateness in focus assignment will give rise to larger brain activity (e.g., P600). These findings suggest that, like topichood, focus devices can increase the prominence of a focused referent. However, unlike focused entities, which are assumed to provide new/unshared information, topic entities encode given/shared information between the interlocutors. The similar pattern of ERP results obtained with these two different information-structural devices suggests that pronoun is preferentially interpreted as referring to prominent entities in terms of information structure, regardless of whether it was assigned by topic or focus devices. This similarity can be accounted for by the accessibility theory (Ariel, 1988, 1990), which assumes that pronouns are resolved toward the most cognitively salient referent in the discourse. In accordance with this approach, although the linguistic functions of topic and focus may be different (e.g., given vs. new), both devices could enhance the salient or prominent status of a referent, and consequently, both render the referent more accessible for a subsequent pronoun (Almor, 1999; Cowles et al., 2007b; Foraker and McElree, 2007; Kaiser, 2011), leading to decreased processing efforts.

## Conclusion

In this study, we manipulated the information status of a referent in terms of pragmatic as well as structural constraints and found that pronouns referring to the referent which encoded topichood evoked an attenuated P600 responses compared to pronouns referring to the referent which encoded subjecthood. Furthermore, P600 was not modulated as a function of whether the [subject] referent was the first- or second-mentioned in the preceding clause. These findings suggest that pragmatically encoded information status, such as topichood, can significantly increase the salience of a referent, whereas word order only exerts limited effects upon on-line pronoun interpretation.

## Conflict of Interest Statement

The author declares that the research was conducted in the absence of any commercial or financial relationships that could be construed as a potential conflict of interest.

## Footnotes

1 OSV structures can occur without this pause even in a non-topic language like in English, but in this case the object is often contrastive, and is serving as a focus rather than a topic. For example, “*I haven’t seen Jack, haven’t seen John either…, MARY I have seen.*”

2 Some Chinese linguists (e.g., Xu and Liu, 2007) argued that the second NP in SOV structure can be treated as second topic of the sentence.

3 Chinese topic structure has been particularly interpreted by using TP (Topic phrase) rather than CP (complementizer phrase) structures, since Chinese has no lexical complementizers without semantic content like *that* in English (Xu, 2000; Xu and Liu, 2007).

4 According to X-bar theory in transformational grammar, an inflection phrase (IP) is a functional phrase that has inflectional properties (such as tense and agreement). An inflectional phrase is essentially the same as a sentence, but reflects an analysis whereby a sentence can be treated as having a head, complement, and specifier.
